# Fe_3_O_4_@PDA/MIL‐101(Cr) as magnetic solid‐phase extraction sorbent for mycotoxins in licorice prior to ultrahigh‐performance liquid chromatography‐tandem mass spectrometry analysis

**DOI:** 10.1002/fsn3.2832

**Published:** 2022-03-21

**Authors:** Zhentao Tang, Qingrong Han, Gang Yu, Fei Liu, Yuzhu Tan, Cheng Peng

**Affiliations:** ^1^ 118385 Key Laboratory of Southwestern Chinese Medicine Resources Innovative Institute of Chinese Medicine and Pharmacy Chengdu University of Traditional Chinese Medicine Chengdu China; ^2^ 118385 Key Laboratory of Southwestern Chinese Medicine Resources School of Pharmacy Chengdu University of Traditional Chinese Medicine Chengdu China; ^3^ Technology Center of Chengdu Customs District P.R. China Chengdu China

**Keywords:** licorice, magnetic metal–organic framework, magnetic solid‐phase extraction, modified QuEChERS, mycotoxins, ultrahigh‐performance liquid chromatography‐tandem mass spectrometry

## Abstract

Magnetic solid‐phase extraction (MSPE) strategy based on the Fe_3_O_4_@PDA/MIL‐101(Cr) has been proposed to separate and purify five common mycotoxins in licorice, including aflatoxin B_1_, aflatoxin G_1_, sterigmatocystin, zearalenone, and ochratoxin A. Integrating the MSPE and solid–liquid extraction/partitioning, a modified QuEChERS was established to adapt to the complex licorice samples. The Fe_3_O_4_@PDA/MIL‐101(Cr) was successfully synthesized and characterized by Fourier transform infrared spectroscopy (FT‐IR), scanning electron microscopy (SEM), transmission electron microscopy (TEM), and nitrogen adsorption–desorption isotherms. Sorbents with superior advantages for exclusion of matrix interference and extraction of target analytes in a short time were obtained, according to their ability of magnetic separation, high surface area (287.75 m^2^/g), large pore volume (0.61 cm^3^/g), and nanosized structure with mesopores. Prior to analysis with ultrahigh‐performance liquid chromatography‐tandem mass spectrometry (UHPLC‐MS/MS), several key parameters that would affect the sorbents’ extraction efficiency were extensively investigated. Under the optimized conditions, the practicality of the developed method for analysis of mycotoxins in licorice samples was confirmed by adequate linearity (*R* ^2^ ≥ 0.9967), high sensitivity (LODs and LOQs, respectively, in the ranges 0.01–0.09 and 0.02–0.30 μg/kg), acceptable recovery (78.53%–116.28%), satisfactory reusability, and good interbatch precision of the sorbents (RSDs in the ranges 6.70%–11.20% and 6.02%–10.35%, respectively). The results indicated that the established method was feasible and reliable for the environment‐friendly and rapid screening of mycotoxins in complex licorice samples.

## Graphical abstract

For the determination of mycotoxins in complex licorice samples, an effective and convenient magnetic solid‐phase extraction (MSPE) was developed based on Fe3O4@ PDA/MIL‐101(Cr). Integrating the MSPE and solid–liquid extraction/partitioning, a modified QuEChERS was established as the sample preparation method. The method coupled to UHPLC‐MS/MS has been demonstrated to provide a feasible, accurate, and rapid method for the determination of mycotoxins in licorice, accordingly providing great potential for analysis of multimycotoxins in other complex matrices.

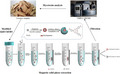

## 1 INTRODUCTION

Licorice is the root of *Glycyrrhiza uralensis* Fisch. or *Glycyrrhiza glabra* L., Leguminosae, which is one of the ancient and worldwide popular herbs native to southern Europe and parts of Asia. With multifunctional ingredients, licorice has beneficial applications in both fields of food and medicine (Peng et al., 2021). It has been on the list of homologous materials of medicine and food with great value, according to the National Health Commission of China. For the value as food, licorice was mainly used in confectionery sectors to produce candies due to the sweet‐tasting compound (Herrera et al., 2009). As for medicinal aspects, licorice has been regarded as the “guide drug” in many traditional Chinese medicine (TCM) prescriptions for thousands of years in China (Wang et al., 2013). Meanwhile, the history of its therapeutic applications has been documented in Europe (Fiore et al., 2005). With the exploitation of its extracts and active ingredients, licorice is extensively applied in food additives, health supplements, food flavoring agents, and cosmetics, more than sweets, herbal remedies, and pharmaceuticals (Hayashi & Sudo, 2009). However, the quality and safety of licorice has encountered the challenge of mycotoxin contamination, which is a common problem in food safety (Fung et al., 2018). Licorice is susceptible to mycotoxin contamination since licorice belongs to crops and can be easily infected by toxigenic fungi during postharvest handling, transportation, and storage processes (Atyn & Twaruek, 2020).

Mycotoxins are a heterogeneous group of toxic metabolites mainly produced by filamentous fungi species of Aspergillus, Penicillium, Alternaria, and Fusarium (Palumbo et al., 2020). They are stable and hard to remove or destroy during the general cooking process. Until now, more than 400 mycotoxins have been discovered with great structural and toxic diversity, some of which are of great concern in food safety, such as aflatoxins (AFs), ochratoxin A (OTA), and zearalenone (ZEN). As is well known, the exposure of mycotoxins to human beings and animals could induce neurotoxic, carcinogenic, nephrotoxic, immunosuppressive, and estrogenic effects (Lee & Ryu, 2016). The existence of mycotoxins in food and agricultural commodities has been recognized as an adverse threat to human health and the economy. Therefore, it is of great importance to determine and monitor the contamination levels of mycotoxins in licorice. With multiple chemical constituents, licorice has an extremely complex matrix, which increases the difficulty of mycotoxin analysis (Li et al., 2016; Shakeri et al., 2018). To be specific, challenges mainly come from the interfering effect on extraction and purification efficiency by starches and polysaccharides as well as the matrix effect, which is defined as suppression or enhancement of the analyte response induced by the coextracted flavonoids, organic acids, or volatile oils (Wei et al., 2018).

Currently, liquid chromatography‐tandem mass spectrometry (LC‐MS/MS) has been widely used as a confirmatory method for mycotoxin analysis on account of its high sensitivity and compatibility with almost the whole range of compound polarities (Dominguez et al., 2020; He et al., 2019; Li et al., 2013). However, the application has been blocked by the matrix effect, which could impact quantification results of the LC‐MS/MS approach, especially for samples with complex matrices. Therefore, reliable and accurate quantification of mycotoxin is subject to efficient sample preparation (Capriotti et al., 2019; Cho et al., 2019). As a critical procedure in instrumental analysis, appropriate sample extraction and clean‐up are effective in reducing the matrix interferences with enrichment of target analytes. Immunoaffinity column and QuEChERS have been widely used as sample preparation methods for mycotoxin analysis (Wei et al., 2018; Zhang et al., 2018). Nevertheless, limitations of expensive cost, cross‐reactivity, low recovery, or sensitivity to matrix types have restricted their use in some cases. As a quick, cheap, and efficient method in analytical chemistry, dispersive solid‐phase extraction (d‐SPE) has gained substantial attention in the analysis of mycotoxins in complex matrices (Jiang et al., 2017; Ran et al., 2017; Reinholds et al., 2019; Tanveer et al., 2020). Especially with the development of novel sorbents, magnetic sorbents are considered in combination with d‐SPE, which is called magnetic solid‐phase extraction (MSPE). MSPE can further simplify the d‐SPE process and is a powerful tool for the application of micro/nanomaterials in sample preparation (Maya et al., 2017). Recently, metal–organic frameworks (MOFs) combined with magnetic nanoparticles have emerged as valuable sorbents for d‐SPE due to their ultrahigh porosity, enormous surface area, tunable pore size, and the ability to realize functionalization and magnetic separation (Gao et al., 2020). Like other advanced materials, MOFs have been widely used for the determination of heavy metals, pesticides, and some other food and environmental pollutants. However, relatively limited research has been conducted to meet the challenge of the determination of trace‐level mycotoxins in complex matrices. Comparatively, mycotoxin analysis with advanced materials as d‐SPE sorbents is rather a new topic (Han et al., 2017; Li et al., 2021; Zhao et al., 2020). Challenges might ascribe to complex sample matrices, trace‐level analytes, and diverse physicochemical properties of mycotoxins. Currently, improvements in mycotoxin analysis have been presented by the application of some advanced sorbents, such as carbon nanotubes, metallic nanoparticles, and graphene‐based materials. It deserves to further investigate the fabrication of advanced sorbents and their application in mycotoxin analysis.

Comprehensively considering the property of mycotoxins and the mechanism of adsorption interaction of MOFs (Hasan & Jhung, 2015; Joseph et al., 2019), this study aims to synthesize Fe_3_O_4_@ PDA/MIL‐101(Cr) and evaluate its performance in the MSPE procedure for analysis of five mycotoxins with high‐frequency contamination rate in licorice. Besides the typical advantages of MOFs, MIL‐101(Cr) is one of the most prominent sorbents owing to its attractive feature of good water stability. To the best of our knowledge, no MSPE method based on magnetic MOFs has been established for the separation and purification of mycotoxins in herbs. Due to the extremely complex matrix of licorice, a modified QuEChERS was developed for sample pretreatment, integrating a solid–liquid extraction/partitioning and the MSPE. Emphatically, MSPE strategy was established by optimization of the relative parameters in detail. Combined with ultrahigh‐performance liquid chromatography‐tandem mass spectrometry (UHPLC‐MS/MS), the developed method was validated and applied in real samples to verify its feasibility.

## 2 MATERIALS AND METHODS

### 2.1 Chemicals and reagents

Standards of aflatoxin B_1_ (AFB_1_), aflatoxin G_1_ (AFG_1_), sterigmatocystin (STER), zearalenone (ZEN), and ochratoxin A (OTA) were supplied by Romer Labs (Beijing) Co., Ltd. The purity of standards was declared in the range 95%–98.9%. The standards dissolved in acetonitrile were stored at −20℃ in amber glass vials.

Acetonitrile (ACN, HPLC grade), methanol (MeOH, HPLC grade), formic acid (MS grade), and ammonium acetate (MS grade) were purchased from Merk Corp. Anhydrous magnesium sulfate, trisodium citrate dihydrate, disodium hydrogen citrate sesquihydrate, sodium chloride, sodium acetate, chromium nitrate nonahydrate, and terephthalic acid were from Aladdin Co. Absolute ethyl alcohol, N, N‐dimethylformamide, hydrochloric acid, and ammonium hydroxide were from Chengdu Kelin Chemical Industry Co. Deionized water was prepared by a Milli‐Q system (Millipore Co.).

### 2.2 Instrument conditions

In this study, the separation and determination of target mycotoxins were performed on an ACQUITY ultrahigh‐performance liquid chromatography (UPLC) system (Waters Corp.) coupled to a Waters Xevo TQ‐XS tandem quadrupole mass spectrometry. The chromatographic separation was realized by an ACQUITY UPLC^®^ BEH C18 column (100 × 2.1 mm, 1.7 μm, Waters Corp.) protected by a VanGuard™ precolumn (5 × 2.1 mm, 1.7 μm, Waters Corp.). The mobile phase consisted of (A) water (containing 5 mM ammonium acetate and 0.1% aqueous ammonia) and (B) methanol. Analyses were carried out with a gradient elution as follows: 10%–60% B at 0–2 min, 60%–90% B at 2–7 min, 90% B at 7–9 min, 90%–10% B at 9.01 min, and 10% B at 9.01–11 min. The injection volume was 5 μl. The column temperature and flow rate were, respectively, set at 40℃ and 0.3 ml/min.

The mass spectrometry was equipped with an electrospray ionization source (ESI). Nitrogen was used as cone, nebulization, and desolvation gas. The MS system was operated with optimal parameters as follows: the source temperature and desolvation gas temperature were, respectively, set at 150 and 250℃. The cone gas flow was maintained at 150 L/h, and desolvation gas flow was 600 L/h. Multiple reaction monitoring (MRM) in positive mode (ESI^+^) was developed for qualification and quantification of target mycotoxins. For each mycotoxin, one precursor ion and two fragment ions were monitored. The cone voltage and capillary voltage of mycotoxins were optimized separately. The optimal MS parameters for analysis of target mycotoxins were listed in Table 1.

**TABLE 1:** Optimized MS/MS parameters of target mycotoxins

Mycotoxin	Precursor ion (m/z)	Product ion 1 (m/z)	Product ion 2 (m/z)	Cone Voltage (V)	CE 1 (V)^b^	CE 2 (V)^b^
AFB_1_	313.10	241.10^a^	285.10^b^	25	50	30
AFG_1_	329.10	243.10^a^	311.10^b^	20	33	20
STER	325.16	281.10^b^	310.11^a^	42	36	24
ZEN	319.25	187.11^a^	283.20^b^	12	18	12
OTA	404.20	221.00^b^	239.30^a^	10	35	30

^a^ Ion pair transition used for qualification;

^b^ ion pair transition used for quantification; and

^b^ collision energy.

Fourier transform infrared spectroscopy (FT‐IR, Thermo Nicolet Corp.), scanning electron microscopy (SEM, Hitachi Co.), transmission electron microscope (TEM, JEOL Ltd.), and specific surface area and porosity analyzer (Micromeritics Corp.) were applied to characterize the prepared sorbent.

### 2.3 Synthesis and characterization of Fe_3_O_4_@PDA/MIL‐101(Cr)

Initially, Fe_3_O_4_ was prepared by the solvothermal method according to the reference (Shao et al., 2012). Fe_3_O_4_@PDA was subsequently prepared by modification of Fe_3_O_4_. Briefly, 0.36 g trismetyl‐aminomethane and 57 μl HCl were dissolved in 300 ml deionized water. Three‐hundred and sixty milligram of Fe_3_O_4_ and 446 mg dopamine were added in the solution and ultrasonicated, followed by being stirred under N_2_ atmosphere for 12 h. After being separated by magnetism, the obtained Fe_3_O_4_@PDA was washed with water and dried under vacuum. With the prepared Fe_3_O_4_@PDA, Fe_3_O_4_@PDA/MIL‐101(Cr) was fabricated as follows: firstly, Fe_3_O_4_@PDA (50 mg), Cr (NO_3_)_3_.9H_2_O (300 mg), terephthalic acid (41 mg), and sodium acetate (450 mg) were dissolved in 40 ml deionized water. The mixture was then transferred into a Teflon‐lined bomb, sealed, and kept at 210℃ for 10 h. After the reaction, the bomb was naturally cooled down to room temperature. The obtained material was purified by N, N‐dimethylformamide and hot ethanol to remove the unreacted terephthalic acid. Finally, the obtained Fe_3_O_4_@PDA/MIL‐101(Cr) was dried under vacuum. Before being used as the MSPE sorbent, the Fe_3_O_4_@PDA/MIL‐101(Cr) was ultrasonicated in methanol and dried.

To characterize the prepared Fe_3_O_4_@PDA/MIL‐101(Cr), Fourier transform infrared spectroscopy, scanning electron microscopy, transmission electron microscopy, and specific surface area and porosity analyzer were applied to investigate the functional groups, morphology, and porous structure, respectively.

### 2.4 Sample collection and pretreatment

Licorice samples were randomly collected from local pharmacies in Chengdu, China. All samples were separately ground and stored in sealed plastic bags below 4°C for further use. The blank samples were confirmed to be free of the target mycotoxins with the analytical method proposed in this work.

Sample powder (2.0 g) was weighed accurately in a 50 ml centrifuge tube. In a typical run, the sample was ultrasonicated for 30 min and centrifuged after being macerated with 20 ml acetonitrile/water (84/16, v/v) for 5 min (Han et al., 2010; Jiang et al., 2017; Zheng et al., 2014). To improve the partition of the mycotoxins into the organic phase, the obtained extraction solution was mixed vigorously for 30 s with 4 g anhydrous magnesium sulfate, 1 g anhydrous sodium chloride, 1 g trisodium citrate dihydrate, and 0.5 g disodium hydrogen citrate sesquihydrate, followed by another centrifugation. Afterward, 5 ml of the resulting ACN‐based supernatant was diluted with ultrapure water to decrease the concentration of organic solvent to 50%. Besides, the pH value of the solution was adjusted to 5.

Twenty milligram of the synthesized Fe_3_O_4_@PDA/MIL‐101(Cr) was added into the sample solution obtained as abovementioned. After being vortexed for 30 min, the mixture was separated by magnetism. Then, the supernatant was discarded, followed by desorption procedure, which was carried out with the following processes: 1 ml acetonitrile containing 2% formic acid was added into Fe_3_O_4_@PDA/MIL‐101(Cr) and the mixture was vortexed for 2 min. With magnetic separation, the supernatant was collected and nearly dried under a mild stream of nitrogen at 35 ℃. The residue was re‐dissolved with 0.5 ml methanol/water (1:1, v/v). After filtering through a 0.22 μm syringe filter, the filtrate was ready for UHPLC‐MS/MS analysis.

## 3 RESULTS AND DISCUSSION

### 3.1 Characterization of the synthesized Fe_3_O_4_@PDA/MIL‐101(Cr)

To verify the functional group of the synthesized Fe_3_O_4_@PDA/MIL‐101(Cr), Fourier transform infrared spectra (FT‐IR) was obtained in the frequency of 400–4000 cm^−1^ for Fe_3_O_4_, Fe_3_O_4_@PDA, and Fe_3_O_4_@PDA/MIL‐101(Cr). As shown in Figure S1, differences in the characteristic functional groups were evident for the three materials. Compared to Fe_3_O_4_, the Fe_3_O_4_@PDA showed unique absorption bands near 1290 and 1500 cm^−1^, of which the former was related to bending vibration of C‐N and the latter for stretching vibration of the aromatic ring. Stretching and bending vibration of the aromatic ring were at 1600 and 1500 cm^−1^, respectively. The characteristic peak of Fe‐O‐Fe was at 590 cm^−1^. The peak at 630 cm^−1^ for Fe_3_O_4_@PDA was ascribed to the vibration of N‐H. With respect to Fe_3_O_4_@PDA/MIL‐101(Cr), the split peak around 1600 cm^−1^ indicated the existence of double carboxyl bonding to the aromatic ring. Symmetric and asymmetric stretching of COO‐ were, respectively, assigned to the peaks near 1400 and 1600 cm^−1^. In addition, the characteristic peak of Cr‐O was at 660 cm^−1^ and close to the adsorption peaks of N‐H and Fe‐O‐Fe, inducing a broad and strong absorption band near 600 cm^−1^. The result demonstrated the successful fabrication of Fe_3_O_4_@PDA/MIL‐101(Cr).

**FIGURE 1:**
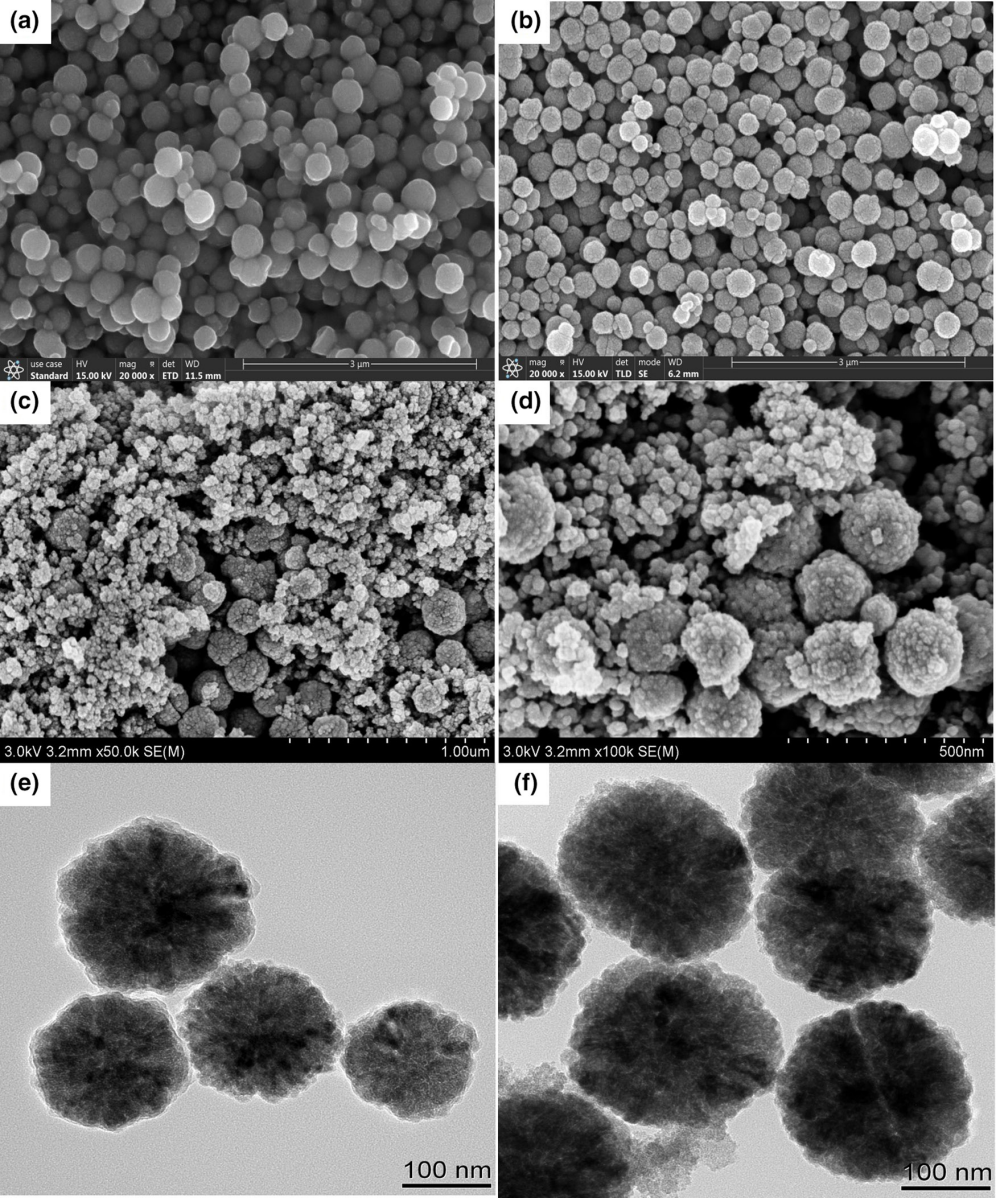
Morphology of the synthesized Fe_3_O_4_, Fe_3_O_4_@PDA, and Fe_3_O_4_@PDA/MIL‐101(Cr), including SEM images of (a) Fe_3_O_4_, (b) Fe_3_O_4_@PDA, and (c and d) Fe_3_O_4_@PDA/MIL‐101(Cr) and TEM images of (e) Fe_3_O_4_@ PDA and (f) Fe_3_O_4_@PDA/MIL‐101(Cr)

The morphology of Fe_3_O_4_@PDA/MIL‐101(Cr) was investigated by scanning electron microscopy (SEM, Figure 1c and d) and transmission electron microscopy (TEM, Figure 1f). For comparison, the SEM image of Fe_3_O_4_ (Figure 1a) and the SEM and TEM images of Fe_3_O_4_@PDA (Figure 1b and e) were examined, respectively. As can be seen from the surface morphology, nanoscale‐sized MIL‐101(Cr) was extended around Fe_3_O_4_@PDA. The obtained Fe_3_O_4_@PDA/MIL‐101(Cr) had a rough and porous structure, which implied a large surface area and high adsorption capacity. On the other side, the TEM image of Fe_3_O_4_@PDA/MIL‐101(Cr) (Figure 1e) showed that MIL‐101(Cr) was randomly grown in the presence of the magnetic particles (Fe_3_O_4_@PDA), further illustrating the extension of MIL‐101(Cr) around the Fe_3_O_4_@PDA.

Furthermore, the porous structure of Fe_3_O_4_@PDA/MIL‐101(Cr) was confirmed by nitrogen adsorption–desorption isotherms. As shown in Figure S2, the Fe_3_O_4_@PDA/MIL‐101(Cr) exhibited a type IV curve with a small hysteresis, indicating the typical property of a mesoporous structure. The Brunauer–Emmett–Teller (BET) surface area of the Fe_3_O_4_@PDA/MIL‐101(Cr) was 287.75 m²/g. And total pore volume was calculated to be 0.61 cm³/g by the Barrett–Joyner–Halenda method. The average pore diameter of the amorphous nanomaterial was about 11.40 nm. The results have verified that the synthesized Fe_3_O_4_@PDA/MIL‐101(Cr) had high surface area, large pore volume, and narrow pore diameter, which was beneficial for its adsorption efficiency and selectivity of analytes from the complex matrix.

### 3.2 Optimization of MSPE procedure

**FIGURE 2:**
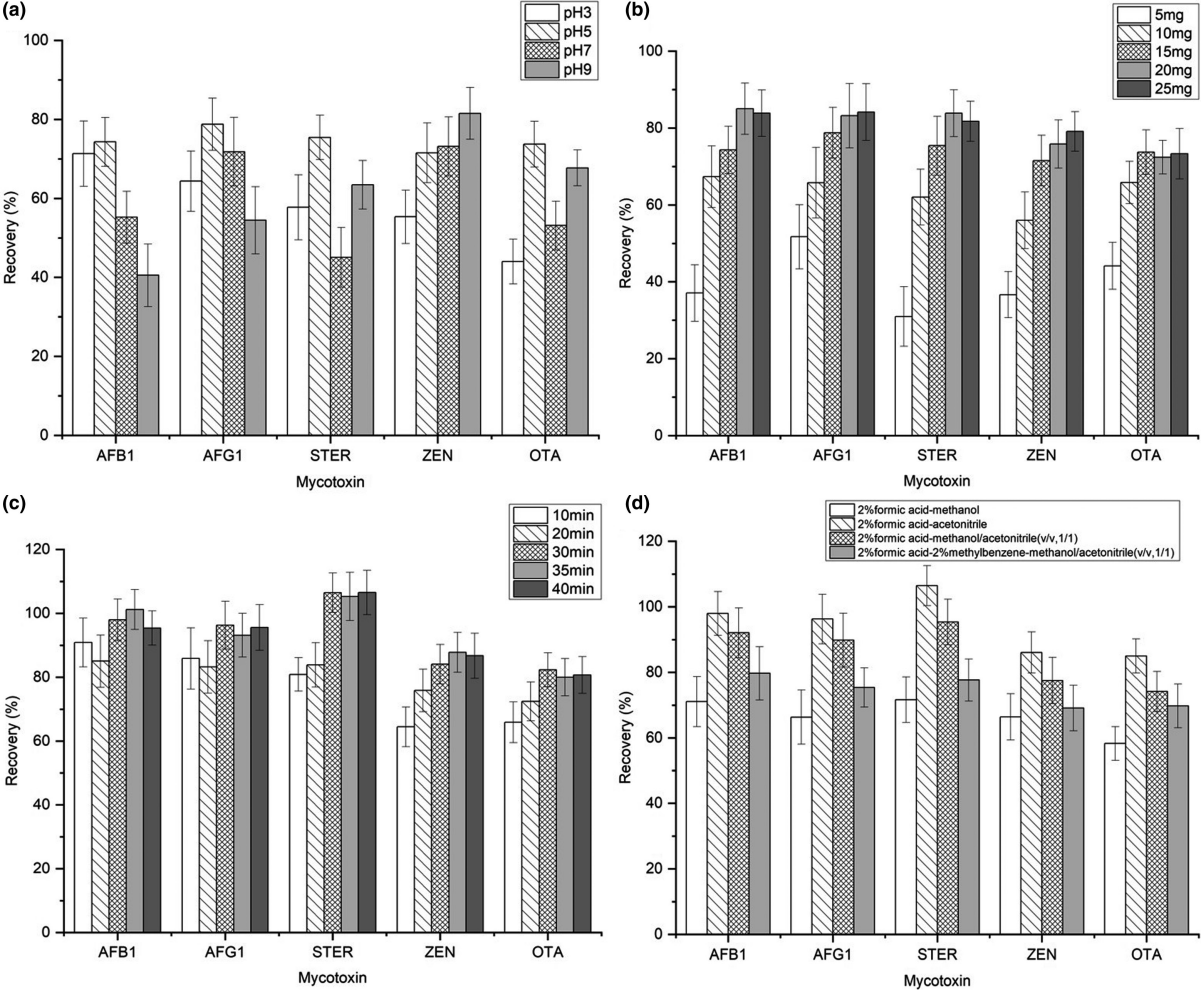
Effect of (a) pH, (b) sorbent amount, (c) adsorption time, and (d) desorption solvent on the recovery (%) of target mycotoxins

To achieve an optimal separation and purification procedure, experimental parameters that could influence the extraction and desorption efficiency of the target mycotoxins were investigated, including pH of the sample solution, sorbent amount, adsorption time, desorption solvent, and desorption time (Figure 2). To take full advantage of the synthesized nanomaterial, all experiments were conducted in triplicate with blank sample extract spiked with target mycotoxins (spiked concentration: 10 μg/L). Diluted hydrochloric acid and ammonium hydroxide were applied to adjust the pH of the sample extracts.

#### 3.2.1 Effect of pH and adsorption mechanism

The pH of the sample solution would determine the existing forms of the analytes as well as the surface‐binding sites of the sorbent. As a consequence, pH has a significant influence on the extraction efficiency of the target mycotoxins. In this study, the effect of pH was investigated in the range from pH 3 to pH 9. In optimization of pH, other parameters were as follows: sorbent amount of 15 mg; adsorption time of 20 min; desorption solvent acetonitrile (2% formic acid); and desorption time of 5 min. As shown in Figure 2a, the highest recovery rates were obtained at pH 5, except for ZEN. Increasing with the pH value, ZEN would lose protons and become negative. The increased recovery of ZEN might be attributed to the electrostatic attraction between the negatively charged analyte and the unsaturated Cr (III) sites of the sorbent. Thus, the main adsorption mechanism of ZEN on the sorbent could be speculated to be electrostatic interaction more than π‐π stacking. As for STER and OTA, recovery increased from pH 3 to pH 5, while obviously decreased from pH 5 to pH 7, and increased again at pH 9. Considering the pKa value (8.38, Figure S3) of STER, the analyte will be in neutral form with good hydrophobicity below pH 6. In this way, high recovery was obtained due to the hydrophobic effect. However, protons would accumulate on the analyte under highly acidic conditions. Thus, poor adsorption recovery was obtained at pH 3 owing to the electrostatic repulsion between the analyte and the sorbent. It is speculated that the main adsorption mechanism of STER might be hydrophobic effect or hydrogen bonding in acidic conditions, while electrostatic interaction in alkaline conditions. In consideration of the pKa values of OTA, its adsorption behavior in the investigated pH range was speculated to be the result of the synergistic effect of electrostatic interaction and hydrogen bonding. As for AFB_1_ and AFG_1_, speculation of the adsorption mechanism was dissimilar to that of other mycotoxins due to the fact that no dissociation equilibrium exists for the analytes. Nevertheless, the structures of aflatoxins have oxygen atoms with lone electrons, which would coordinate with H^+^ in highly acidic conditions. Then, poor recovery was similarly obtained at pH 3 for electrostatic repulsion. Theoretically, the adsorption of AFB_1_ and AFG_1_ was possibly driven by π‐π stacking, hydrogen bonding, and hydrophobic effect. When the pH value was more than 5, the carboxyl group on the sorbent was deprotonated along with the hard formation of hydrogen bond between the analyte and the sorbent, thus the adsorption recovery was relatively decreased. In comprehensive consideration, pH 5 was employed in subsequent tests for appropriate recovery rates.

#### 3.2.2 Effect of sorbent amount

The sorbent amount was evaluated in the range 5–25 mg. As shown in Figure 2b, it revealed that the sorbent amount had a significant impact on the recovery of target mycotoxins, especially when the sorbent amount was increased from 5 to 15 mg. In optimization of the sorbent amount, other parameters were as follows: pH 5; adsorption time of 20 min; desorption solvent acetonitrile (2% formic acid); and desorption time of 5 min. According to the result, the recovery of five target mycotoxins was obviously increased, with the sorbent amount varying from 5 to 15 mg. In comparison with the experiment using 15 mg of sorbent, an experiment with 20 mg of sorbent resulted in better recovery for AFB_1_, AFG_1_, STER, and ZEN. As for OTA, a further increase in the sorbent amount nearly had no influence on its recovery. Furthermore, it was shown that 25 mg of sorbent induced a slight increase on the recovery of ZEN, while no obvious change on the recovery of other mycotoxins. Consequently, 20 mg of sorbent was a reasonable compromise to ensure satisfactory extraction efficiency and recovery.

#### 3.2.3 Effect of adsorption time

As the adsorption process is an equilibrium‐based extraction procedure, adsorption time is another significant factor to impact the extraction efficiency and recovery of the mycotoxins. In this study, vortex mixing was employed in the adsorption process to facilitate the achievement of adsorption equilibrium. In optimization of the adsorption time, other parameters were as follows: pH 5; sorbent amount of 20 mg; desorption solvent acetonitrile (2% formic acid); and desorption time of 5 min. From the result in Figure 2c, the equilibrium of target mycotoxins was almost reached at an adsorption time of 30 min. As the adsorption time further increased, the recovery of target mycotoxins remained nearly the same. Therefore, 30 min was chosen as the optimal adsorption time.

#### 3.2.4 Effect of desorption solvent and time

To guarantee satisfactory desorption efficiency of the analytes from sorbent, desorption solvent and desorption time have been, respectively, investigated in this study. In optimization of the desorption procedure, other parameters were as follows: pH 5; sorbent amount of 20 mg; and adsorption time of 30 min.

According to the result of the pre‐experiment, the addition of formic acid in desorption solvent could increase the elution efficiency of target mycotoxins in this study. Therefore, four kinds of desorption solvents were specifically considered, including 2% formic acid—methanol, 2% formic acid—acetonitrile, 2% formic acid—methanol/acetonitrile (v/v = 1/1), and 2% formic acid–2% methylbenzene—methanol/acetonitrile (v/v = 1/1). The result in Figure 2d indicated that the highest recovery rates were obtained when 2% formic acid–acetonitrile was used as the desorption solvent. Experiments with desorption time of 2 and 5 min were also compared, resulting in no obvious distinction. It is implied that 2 min was enough to obtain satisfactory recovery of the mycotoxins.

### 3.3 Determination of the matrix effect

As a well‐known problem for electrospray ionization, the matrix effect appeared as signal suppression or signal enhancement of analyte is unavoidable due to the co‐elution of matrix interferants. To characterize the matrix effect of the proposed method, matrix‐matched calibration curves were established by the addition of standard solution into the blank sample extract. Simultaneously, solvent calibration curves were established. Matrix effects defined as signal suppression/enhancement (SSE) were then evaluated as the ratio of matrix‐matched calibration slope to solvent calibration slope.

SSE = $\frac{\mathrm{matrix}‐\mathrm{matched}\;\mathrm{calibration}\;\mathrm{slope}\;}{\mathrm{solvent}\;\mathrm{calibration}\;\mathrm{slope}}$ × 100%

From the result in Table 2, the average SSE of AFB_1_, AFG_1_, ZEN, and OTA was in the acceptable range from 75.58% to 90.57%, while the signal of STER was suppressed by 39%. In other words, the purified sample matrix had no significant influence on the response of AFB_1_, AFG_1_, ZEN, and OTA, while having a relatively prominent effect on the response of STER. Considering the fact that matrix‐matched standards are commonly used to compensate matrix effect, matrix‐matched calibration was essential for the accurate determination of target mycotoxins in this study.

**TABLE 2:** Signal suppression / enhancement (SSE) of the proposed method in raw licorice matrix

Mycotoxin	SSE (%)	RSD (%, *n* = 3)
AFB_1_	75.58	9.08
AFG_1_	78.30	10.79
STER	61.06	12.36
ZEN	88.91	7.59
OTA	90.57	6.58

### 3.4 Method validation

To validate the proposed method, parameters that reflect the efficiency and feasibility of the method were estimated by investigating the analytical characteristics in terms of linearity, limits of detection (LODs), limits of quantification (LOQs), the reusability, and interbatch precision of the sorbents. The obtained results were listed in Table 3.

**TABLE 3:** Linear range, correlation coefficients, limits of detection, limits of quantification of the established method, and the reusability and inter‐batch precision of the sorbents for target mycotoxins

Mycotoxin	Linear range (μg/L)^a^	Intercept	Intercept Standard Error	Slope	Slope Standard Error	*R* ^2^	LOD (μg/kg)	LOQ (μg/kg)	Reusability (RSD%, *n* = 5)	Interbatch precision (RSD%, *n* = 3)
AFB_1_	0.5–50	293.43	343.00	2499.57	60.17	0.9983	0.02	0.07	8.76	6.02
AFG_1_	0.5–50	1467.81	329.83	2315.77	57.86	0.9981	0.05	0.18	10.13	8.29
STER	0.5–50	468.94	349.08	1923.35	61.23	0.9969	0.09	0.30	11.20	10.35
ZEN	0.5–50	5138.84	4150.49	21,914.59	728.04	0.9967	0.01	0.02	7.69	6.09
OTA	0.5–50	703.00	787.37	5440.50	138.11	0.9981	0.01	0.04	6.70	6.57

^a^ The concentration levels were 0.5, 1.0. 2.0, 5.0, 10.0, 20.0, and 50.0 μg/L, respectively.

The linearity was studied by spiking a series of standard solution to the blank sample extract over the concentration of 0.5–50 μg/L for AFB_1_, AFG_1_, STER, ZEN, and OTA. A satisfactory linear relationship was finally obtained with the *R* ^2^ values between 0.9967 and 0.9983. The LODs and LOQs were estimated at the lowest concentration of calibration solution as 3 times and 10 times ratio of signal to noise (S/N), respectively. For mycotoxins with regulatory limits in licorice, the LOQs of AFB_1_ and OTA were 0.07 and 0.04 µg/kg, respectively. The obtained LOQs were smaller than MRLs (maximum residue limits) established by the European Union (2 µg/kg for AFB_1_ and 20 µg/kg for OTA) (European Union, 2006, 2009), indicating that the established method would be sufficiently sensitive for determination of target mycotoxins in licorice samples. The reusability of the sorbent was evaluated by five consecutive experiments of the spiked sample extract (spiked concentration: 10 μg/L). In a typical run, the sorbent was collected and washed several times with methanol and deionized water following the desorption procedure. After being dried, the sorbent was reused according to the above‐mentioned adsorption and desorption procedure. The recovery variation expressed in RSDs was in the range 6.70%–11.20%. The interbatch precision was assessed for three different batches of sorbents, with the resulted RSDs smaller than 10.35%. The result demonstrated that the sorbent was stable and reusable enough for the determination of mycotoxins in licorice samples.

To further confirm the accuracy of the proposed method, recovery of target mycotoxins spiked in licorice samples at three different concentration levels was evaluated. Each concentration level was investigated as three replicates of the spiked samples, which were extracted, purified, and analyzed by the established method. Specifically, recovery was calculated as the ratio of the actual response to the theoretical response on the basis of the matrix‐matched calibration. The obtained results were summarized in Table 4. It can be seen from the result that the average recovery of target mycotoxins ranged from 78.53% to 116.28%, with RSDs ranging from 5.58% to 10.77%. The obtained result demonstrated that the established method would provide satisfactory sensitivity, accuracy, and reliability for the determination of target mycotoxins in licorice.

**TABLE 4:** Recovery of target mycotoxins in spiked licorice samples at three different concentrations

Mycotoxin	Low (1.68 μg/kg)		Middle (8.40 μg/kg)		High (16.80 μg/kg)	
	Recovery (%)	RSD (%, *n* = 3)	Recovery (%)	RSD (%, *n* = 3)	Recovery (%)	RSD (%, *n* = 3)
AFB_1_	78.53	6.69	81.85	6.17	85.17	5.58
AFG_1_	80.32	7.32	85.95	6.76	83.39	7.05
STER	107.15	10.77	116.28	10.57	113.30	9.28
ZEN	80.37	8.52	89.22	7.35	90.83	7.77
OTA	85.01	6.99	81.49	6.37	88.58	6.75

### 3.5 Comparison of the proposed method with other methods

**FIGURE 3:**
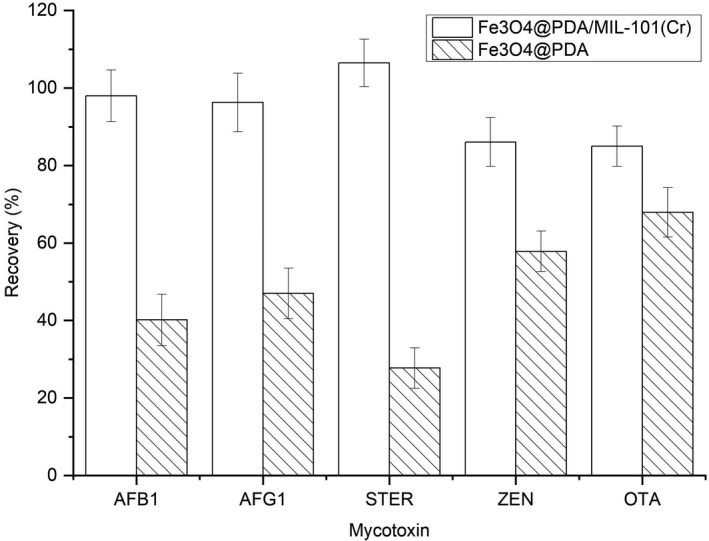
Comparison of the extraction efficiency of target mycotoxins in licorice sample between Fe_3_O_4_@PDA and Fe_3_O_4_@PDA/MIL‐101(Cr)

To evaluate the separation and purification effect of the synthesized nanomaterial, adsorption of the target mycotoxins in the licorice sample was compared between the synthesized Fe_3_O_4_@PDA and Fe_3_O_4_@PDA/MIL‐101(Cr). As presented in Figure 3, Fe_3_O_4_@PDA/MIL‐101(Cr) showed better extraction efficiency for five mycotoxins. In comparison with Fe_3_O_4_@PDA, the more rough and porous structure of the Fe_3_O_4_@PDA/MIL‐101(Cr) could further increase the adsorption capacity of the sorbent. More importantly, the mesoporous structure seems to be beneficial to reducing the interference of sample matrix, especially for macromolecular compounds. Therefore, the synthesized Fe_3_O_4_@PDA/MIL‐101(Cr) has been proved effective for the adsorption of mycotoxins in licorice.

Furthermore, the proposed method has been compared with d‐SPE, SPE, and QuEChERS approach reported for the determination of mycotoxins in herbs by previous references (Han et al., 2012; Jiang et al., 2017; Luo et al., 2018; Ran et al., 2017; Tanveer et al., 2020; Xing et al., 2016). The comparison with MSPE methods for the determination of mycotoxins in some other food was also considered (Han et al., 2017; Li et al., 2021; Zhao et al., 2020). It can be clearly seen from Table 5 that comparable or even better sensitivity of the established method was obtained. Compared to the traditional SPE or d‐SPE, the method established in this article consumed less time and volume of organic solvent for sample pretreatment. C18 was used as disposable purification sorbent in hundreds of milligrams for the QuEChERS method, while 20 mg of Fe_3_O_4_@PDA/MIL‐101(Cr) could be reused at least five times in this work. As for the comparison with other MSPE methods, the method showed some insufficiency in respect of sample pretreatment time. Considering the existence as solid samples and the particularly complicated matrix of licorice, the MSPE procedure has taken full advantage of the synthesized Fe_3_O_4_@PDA/MIL‐101(Cr), which could realize the adsorption and desorption of analytes in less than 35 min with only 6 ml organic solvent. In summary, the developed method exhibited advantages of easy operation, satisfactory sensitivity, good efficiency, environment friendly, and less economic cost.

**TABLE 5:** Comparison of the established method with previously reported methods

Sample	Sample pretreatment	Sorbent	Amount of sorbent (mg)	Volume of organic solvent (mL)	Pretreatment ime	Analytical technique	LOQ (μg/kg)					Recovery (%)	Reference
							AFB_1_	AFG_1_	STER	ZEN	OTA		
Licorice	MSPE	Fe_3_O_4_@PDA/MIL‐101(Cr)	20	18.05	>72.5	UHPLC‐MS/MS	0.07	0.18	0.30	0.02	0.04	78.53–116.28	This study
Maize	MSPE	Fe_3_O_4_/MWCNT	20	19.65	>58	UHPLC‐MS/MS	‐^a^	‐^a^	‐^a^	0.07	‐^a^	100.50–104.10	(Han et al., 2017)
Milk	MSPE	PEG‐MWCNTs‐MNP	10	15.25	>41	UHPLC‐QE‐MS	0.03	0.06	‐^a^	0.15	0.15	81.80–106.40	(Zhao et al., 2020)
Milk	MSPE	PEI@glymo@Fe_3_O_4_MWCNTs	20	26.50	>11	HPLC‐MS/MS	0.01	0.01	0.13	0.15	‐^a^	88.30–103.50	(Li et al., 2021)
Coptidis rhizoma	d‐SPE	rGO‐ZnO	15	25.30	>85.0	UHPLC‐MS/MS	0.09	0.10	‐^a^	0.10	0.10	77.30–103.80	(Tanveer et al., 2020)
PCM	d‐SPE	GO	2.5	20.05	>95.5	HPLC‐FD	0.09	0.13	‐^a^	‐^a^	‐^a^	74.00–102.70	(Ran et al., 2017)
Salviae miltiorrhizae Radix et Rhizoma	SPE	Fe_3_O_4_/MWCNT	20	18.95	>>50.5^a^	UHPLC‐MS/MS	‐^a^	‐^a^	‐^a^	1.60	‐^a^	88.20–91.90	(Jiang et al., 2017)
Rhizomes and roots	SPE	Silica gel	2000	25.56	>>15.0^a^	UHPLC‐MS/MS	0.02	0.09	0.02	0.32	0.04	71.40–119.30	(Han et al., 2012)
Menthae haplocalycis	QuEChERS	C18	100	5.15	>72.5	UHPLC‐MS/MS	0.03	0.03	‐^a^	0.05	0.04	67.10–103.00	(Luo et al., 2018)
Redix Paeoniae Alba	QuEChERS	C18	150	2.75	>14.5	UHPLC‐QqLIT‐MS	0.25	0.39	0.25	0.20	0.50	83.10–104.30	(Xing et al., 2016)

^a^ Apart from the extraction time, there is indefinite time of loading, wash, and elution in SPE process.

^b^ Not tested in the references.

### 3.6 Application to licorice samples

**FIGURE 4:**
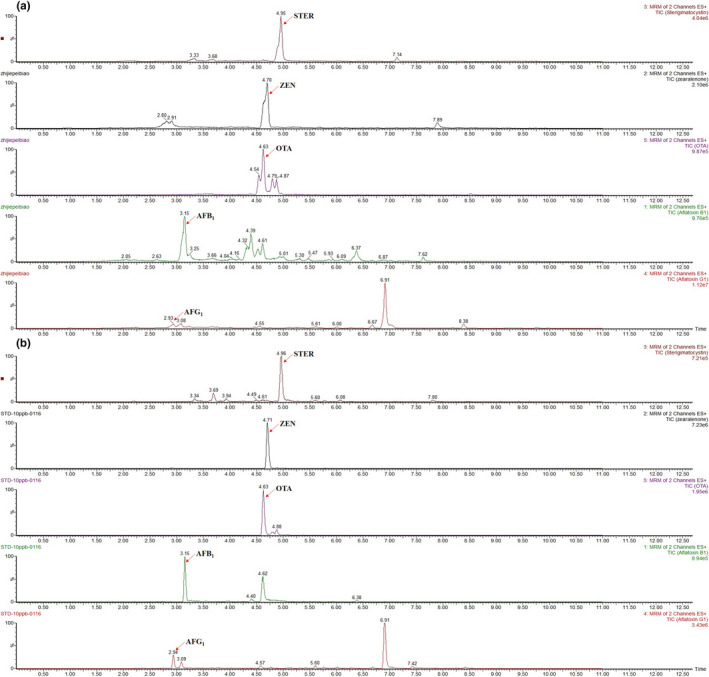
Total ion chromatograms of five mycotoxins in a spiked licorice sample without (a) and with (b) extraction by the synthesized Fe_3_O_4_@PDA/MIL‐101(Cr)

Total ion chromatograms of five mycotoxins in a spiked licorice sample with and without being processed by the Fe_3_O_4_@PDA/MIL‐101(Cr) were shown in Figure 4. After being processed by the salts to enhance the partition of the extracted solution, one part of the resulting ACN‐based supernatant was diluted with water (1:1, v/v) and filtered through a 0.22 μm syringe filter for analysis. And the other part was diluted and treated with the MSPE procedure as described above. It has been clearly shown that the interference of sample matrix has been greatly reduced with the MSPE procedure, which resulted in better chromatographic separation. To further evaluate the applicability of the proposed method, target mycotoxins in five commercial licorice samples were analyzed. The result of real sample analysis showed that no other mycotoxins were detected in five licorice samples, except for OTA. The contamination levels of OTA were below the LOQ value of the method, which was far lower than the MRL established by the European Union (20 µg/kg).

## 4 CONCLUSION

In this study, Fe_3_O_4_@PDA/MIL‐101(Cr) was successfully synthesized and creatively applied as MSPE sorbent for separation and purification of five common mycotoxins in licorice sample extract. A modified QuEChERS based on the MSPE has been proved to possess the efficient capability of simultaneous separation, purification, and enrichment of target mycotoxins from complex licorice samples. Avoiding tedious procedures, the proposed sample pretreatment method could realize the extraction and purification of analytes with relatively little time and organic solvent consumption. Even for licorice samples with quite complicated components, the established method coupled with UHPLC‐MS/MS analysis has been demonstrated to provide a feasible, sensitive, and rapid method for the determination of mycotoxins. With the ability of rapid screening of mycotoxins in licorice samples, the proposed method suggests a potential application for the determination of multimycotoxins in samples with complex matrices. Since a few mycotoxins have been considered in this study, more work is still needed in the following studies.

## CONFLICT OF INTEREST

The authors declare no conflict of interest.

## Supporting information

Fig S1‐S3

## Data Availability

The data that supports the findings of this study are available on request from the authors.
