# Supplementary material for: Fe3O4@PDA/MIL‐101(Cr) as magnetic solid‐phase extraction sorbent for mycotoxins in licorice prior to ultrahigh‐performance liquid chromatography‐tandem mass spectrometry analysis
Source: Food Sci Nutr. 2022 Mar 21;10(7):2224–35. doi: 10.1002/fsn3.2832 (PMC9281945; doi:10.1002/fsn3.2832)

**Supplementary information for**

**Fe_3_O_4_@PDA/MIL-101(Cr) as magnetic solid-phase extraction sorbent for mycotoxins in licorice prior to ultra-high performance liquid chromatography-tandem mass spectrometry analysis**

Zhentao Tang^a^, Qingrong Han^b^, Gang Yu^c^, Fei Liu^c^, Yuzhu Tan^b, *^, Cheng Peng^b, *^

*^a^ Key Laboratory of Southwestern Chinese Medicine Resources, Innovative Institute of Chinese Medicine and Pharmacy,* *Chengdu University of Traditional Chinese Medicine, Chengdu, China*

*^b^ Key Laboratory of Southwestern Chinese Medicine Resources, School of Pharmacy, Chengdu University of Traditional Chinese Medicine, Chengdu, China*

*^c^* *Technology Center of Chengdu Customs District P.R.China, Chengdu, China*

^*^ Corresponding author.

Yuzhu Tan

Mail: Chengdu University of TCM, 1166 Liutai Avenue, Chengdu 611137, China;

E-mail: [tanyuzhu@cdutcm.edu.cn](mailto:tanyuzhu@cdutcm.edu.cn);

Phone: (+86)18683686528.

Cheng Peng

Mail: Chengdu University of TCM, 1166 Liutai Avenue, Chengdu 611137, China;

E-mail: [pc@cdutcm.edu.cn](mailto:pc@cdutcm.edu.cn).

Fig. S1. FT-IR of the synthesized Fe_3_O_4_, Fe_3_O_4_@PDA and Fe_3_O_4_@PDA/MIL-101(Cr).


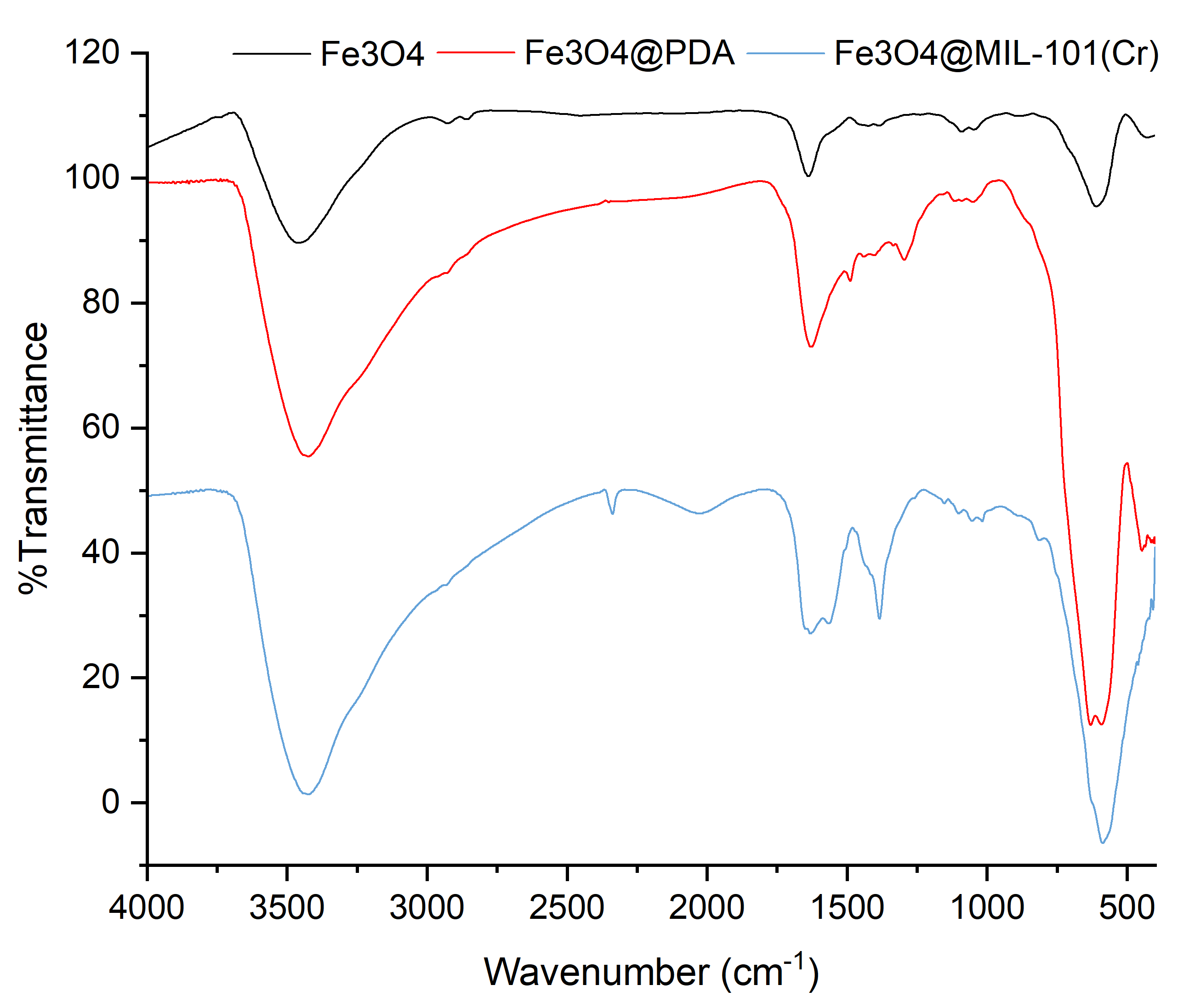


Fig. S2. N_2_ adsorption-desorption isotherm of the Fe_3_O_4_@PDA/MIL-101(Cr).


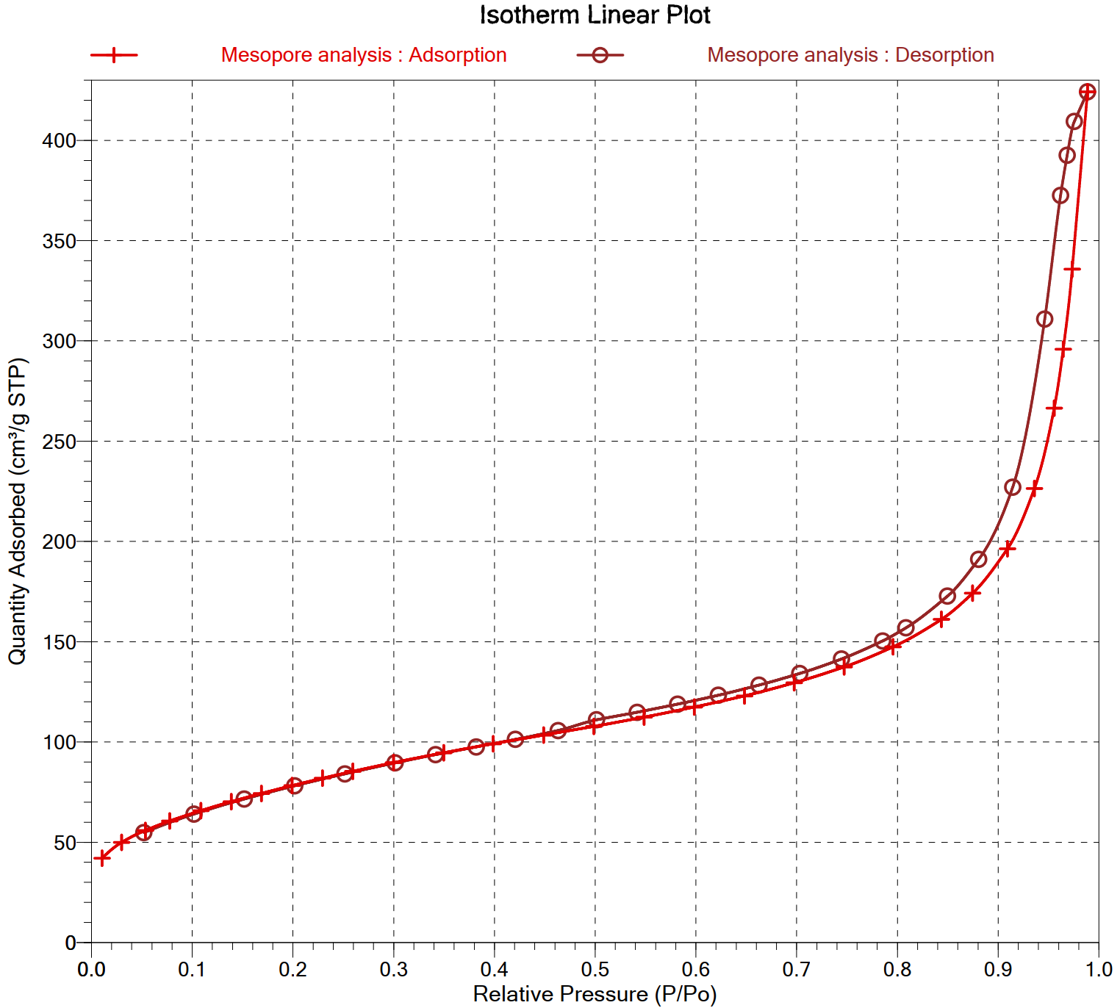


Fig. S3. Dissociation equilibrium constants and distribution curve of STER, OTA and ZEN.


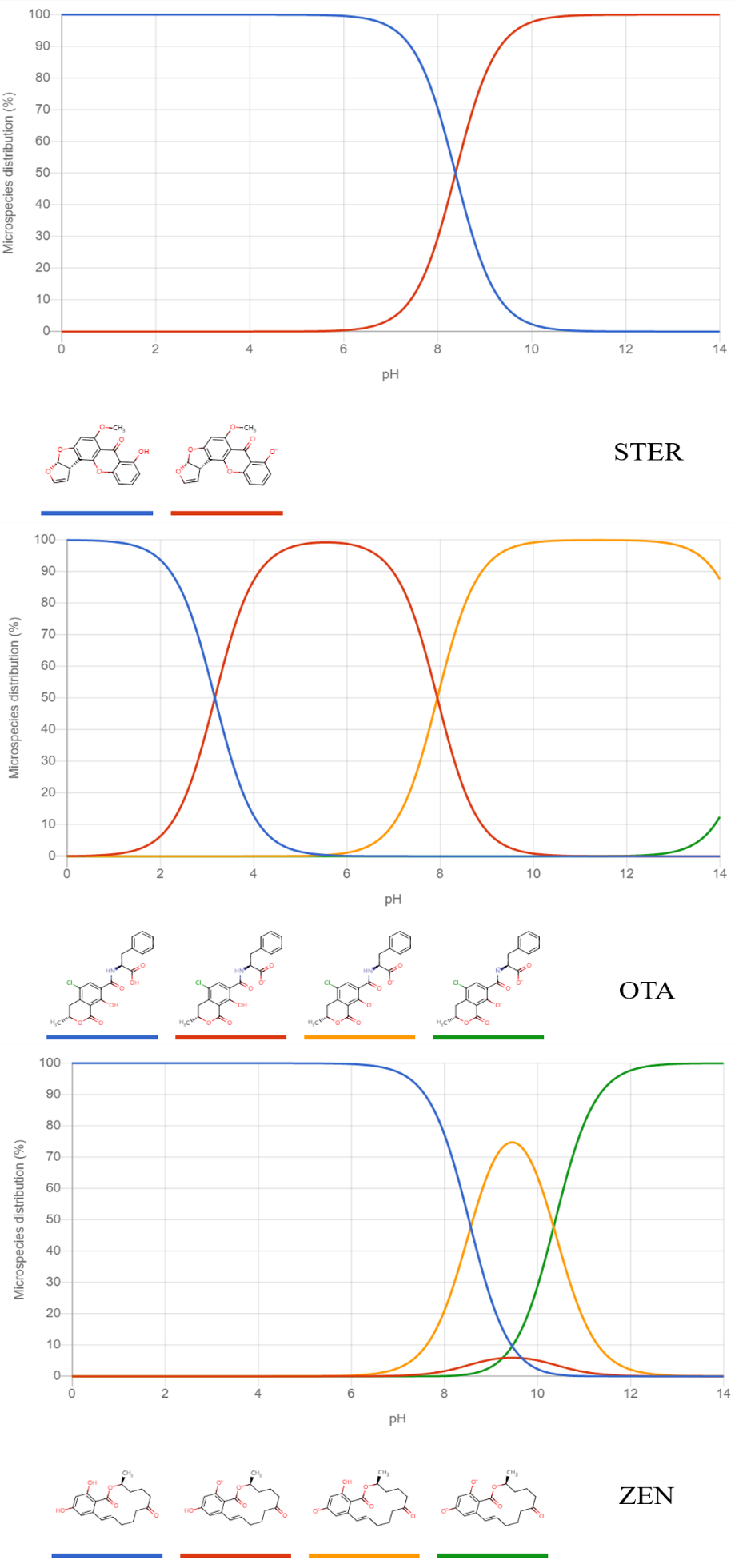

Supplement: Supplementary file 1 — Fig S1‐S3 [file FSN3-10-2224-s001.docx]
